# Distribution of new fluorene disulphonamido derivatives in rats with transplanted Walker carcinosarcoma.

**DOI:** 10.1038/bjc.1966.99

**Published:** 1966-12

**Authors:** D. Malejka


					
857

DISTRIBUTION        OF    NEW     FLUORENE        DISULPHONAMIlDO

DERIVATIVES IN RATS WITH TRANSPLANTED WVALKER
CARCINOSARCOMA

DANUTA MALEJKA*

From the Department of Pharmaceutical Chemistry, Medical Academy,

ul. Grunwaldzka 6, Poznah, Poland

Received for publication June 1, 1966

THE previous investigation by this author dealt with the distribution of two
new fluorene disuiphonamides : N,N' -bis(thiazole)- 2,7 -fluorene[35S ]disulphona-
mide, I, and N,N'-bis(guanidine)-2,7-fluorene[35S]disulphonamide dihydrate, II,
in rats bearing Walker carcinosarcoma and in tumour-free animals (Malejka.
1965). Wihile the bis-thiazole derivative revealed an appreciable concentration
in tumour- 35 atg. per g. tissue (8 per cent of the dose given, the bis-guanidyl
derivative was not found in tumour. In the case of Compound I, autoradiograms
of tumour slides showed a considerable uptake of radioactivity by tumour cells
and particularly by their nuclei (Kasprzak, Malejka, Gabryel, 1965). The
tumour concentration for this conipound appeared, however, not to be highly
selective: the ratios of the radioactivity concentration in tumour to that in
liver or spleen or blood plasma were lower than 0 5. These findings favour the
hypothesis (Argus, 1961) that the presence of free acidic groups in the fluorene
disulphonamido high molecular compounds contributes to their binding at the
cellular adsorption sites by basic proteins.

The other aspect of the diagnostic studies with the fluorene disulphonamido
group was to observe the reaction of the reticulo-endothelial system in the presence
of tumour. The phenomenon of an impaired phagocytic function of the reticulo-
endothelial system was demonstrated in the presence of various tumours in differ-
ent species using N,N'-bis(naphthalene)-2, 7-fluorene[35S]disulphonamide (Argus
and Hewson, 1954; Argus, Hudson, Seepe, Kane and Ray, 1962). The same
reaction was observed with N,N'-bis(p-sulphamoyl-phenyl)-2,7-fluorene[35S]disul-
phonamide (Malejka, 1962). The importance of the precise chemical structure is
shown by the failure of N,N'-bis(thiazole)-2, 7-fluorene[35S]disulphonamide to
localize in liver and spleen of tumour-free animals to a greater extent than in
tumour-bearers (Malejka, 1965).

The present investigation was aimed at studying the influence of various
substituents in the fluorene disulphonamide molecule on the distribution of the
compounds in tumour-bearing and tumour-free rats. Five new derivatives of
35S-labelled 2,7-disulphonamido-fluorene were synthesized in which the substitu-
ents on the nitrogens were chosen so as to permit study of the effect of different
structural types on the tissue localization.

* Present address: Pharmaceutical Chemistry Research Laboratory College of Pharmacy,
University of Florida, Gainesville, Florida, U.S.A.

858

DANUTA MALEJKA

H2N-OC-HN-028      -80j\jf)   SO2-NH-CO-NH2

N,N'-bis(carbamyl)-2,7-fluorene[35S]disulphonamide, III

H9C 4-N-02S                 So2-N-C4H9

I                 7

RN.L 'INH

U2S                           SO2  /H3

N,N'-bis(p-tolylsulphonyl-carbamyl-n-butyl)-2,7-fluorene[35S] disulphonamide, IV

HOOC            HN-02      7N              02-NH    /         COOH

N,N'-bis(4-carboxy-phenyl)-2,7-fluorene[35S]disulphonamide, V

02N                              NO2

N                  7

NNb()n      2        )-        o [S2-N   sh         V

N,N'-bis(5(6)-niitro-benzimnidazolyl)-2,7-fluorene[35S]disulphonamide,, VI

H3CO                                       OCH3

N,N'-bis(6(2,4-dimethoxy-pyrimidyl))-2,7-fluorene[35S]disulphonamide, VII

TRANSPLANTED WALKER CARCINOSARCOMA

Compound III is the condensation product of 2,7-fluorene [35S] disulphonyl-
chloride with 2 molecules of urea, thus representing a simple aliphatic side chain
and showing a structural resemblance to the previously described bis-guanidyl
derivative (Compound II) (Malejka, 1965). The urea moiety occurs also in Com-
pound IV in which 2 molecules of tolbutamide, (l-butyl-3(p-tolyl-sulphonyl)urea).
a hypoglycaemic sulphonamide, have been used to increase the molecular weight
of the new compound. Compound V is an analogue of N,N'-bis(p-carboxy-
phenyl)-4,4'-biphenyl [35S] disulphonamide which in the earlier studies in mice
revealed an appreciable concentration in tumour (Argus, Seepe, Gutierrez, Hewson,
Rav, 1958).

Previous studies with the bis-thiazole derivative (Compound I) emphasized
the possible meaning of heteroatoms in the thiazole rings, particularly electro-
negative sulphur for its ability to bind liver and serum proteins and which might
influence the affinity of this compound for neoplasms (Kasprzak, Malejka, Gabryel,
1965  Malejka, Kasprzak, Radola, 1966). These studies were now extended by
substituting 2,7-disulphonamido fluorene with groupings containing the some-
what more electronegative nitrogen. Two heterocyclic groupings, 5(6)-nitro-
benzimidazolyl and 2,4-dimethoxy-6-amino-pyrimidyl were chosen for this purpose
and the respective condensation products are Compounds VI and VII.

MATERIALS AND METHODS

2.7-Fluorene[35S]disulphonylchloride.-

The procedure described previously (Argus and Hewson, 1954) was employed,
but double batches of material were prepared. Fifty millicuries of 35S (H235SO4)
wAere used to label each portion of chlorosulphonic acid. The yield of crude
material was 25 g. (71 per cent of theory) per batch. Recrystallization from
toluene gave 10-7 g. of the product (30.4 per cent of theory) melting at 219-2210 C.:
specific activity was not determined on this intermediate.

N,N'-bis(carbamyl)-2,7-fluorene[35S]disulphonamide, III.-

Urea, 1.8 g. (0.03 moles) was dissolved in 1-6 ml. of hot water and 10 ml. of
acetone was added. The solution was stirred and maintained at 550 C. (water
bath) throughout the addition (30 minutes) of the suspension of 2,7-fluorene[35S]
disulphonylchloride, 2 g. (0.0055 moles) in 60 ml. of acetone. After refluxing
3 hours the resulting white precipitate was collected and dried over phosphorus
pentoxide. The yield of crude material was 2 g. (91.7 per cent of theory); it
did not melt, but darkened at 2400 C. Purification from 96 ml. of n-butanol
and methanol mixture (2: 1) with charcoal gave 1-3 g. (59.6 per cent of theory)
of cream-coloured crystalline material, darkening at 250? C. (complete decomposi-
tion at 320? C.) and having a specific activity of 2000 disintegrations per second
per mg. (0.054 ,uC per mg.).

Analysis.-Calculated for C15H14N4S206 (molecular weight 410-36) : C. 43 90;
H, 3.44; N, 13-65; S, 15-63. Found: C, 43-72; H, 3-51; N, 13-33.

N,N'-bis(p-tolylsulphonyl-carbamyl-n-butyl)-2,7-fluorene[35S]disulphonamide,

N-p-tolylsulphonyl-N-n-butyl-urea (Tolbutamide, U. S. P.), 6.9 g. (0.025 moles)

859

DANUTA MALEJKA

was dissolved in 15 ml. acetone and methyl-ethyl-ketone mixture (1: 1) at 60 C(.
(water bath). The solution was stirred and maintained at the same temperature
throughout the addition (15 minutes) of the solution of 2,7-fluorene[35S]disulpho-
nylchloride, 3 g. (0.008 moles) in 75 ml. mixture of the same solvents. The reac-
tion mixture was stirred throughout the following 12 hours at 65-70? C. (a cream-
coloured precipitate started to form after 2 hours of stirring). On cooling to
room temperature the precipitate was collected and dried over phosphorus pent-
oxide. The yield of crude material was 5-6 g. (81.2 per cent of theory). melting
point 290? C. Triple recrystallization from absolute methanol (first time with
charcoal) gave 1.45 g. (21 per cent of theory) of white product melting at 313-
3150 C. and having a specific activity of 1200 disintegrations per second per mg.
(0.032 ,uc per mg.).

Analysis.-Calculated for C37H42N4S4010 (molecular weight 835): C, 53 70;
H, 5 07; N, 6*71; S, 13-56. Found: C, 53-42; H, 5-12; N, 6 60.

N,N'-bis(4-carboxy-phenyl)-2,7-fluorene[35S]disulphonamide, V.-

p-Aminobenzoic acid, 3-6 g. (0.027 moles) was dissolved in a mixture of 20 mnl.
acetone and 15 ml. of toluene at 50-60? C. (water bath). The solution was stirred
and maintained at the same temperature throughout the addition (15 minutes)
of solid 2,7-fluorene[35S]disulphonylchloride, 2 g. (0.0055 moles) and throughout
the following 5 hours. The resulting pale pink precipitate was collected, washed
free of chlorides with warm distilled water and then with toluene and dried over
phosphorus pentoxide. The yield of crude material was 2-6 g. (86-6 per cent of
theory) carbonizing at 260? C. Purification by double extraction with hot aniline
(30 ml. and 20 ml.) followed by several washings with hot toluene gave 0.9 g.
(30 per cent of theory of white, fine crystalline material, carbonizing within the
range of 325-350? C. and having a specific activity of 1330 disintegrations per
second per mg. (0.036 ,uc per mg.).

Analysis.-Calculated for C27H20N2S208 (molecular weight 564.61): C. 60.88;
H, 3*79; N, 5-26; S, 12-01. Found: N, 5 40; S, 12-21.

N,N'-bis(5(6)-nitro-benzimidazolyl)-2,7-fluorene[35S]disulphonamide. VI.-

Nitro-5(6)-benzimidazole, 3 g. (0.018 moles) was dissolved in 45 ml. of anhyd-
rous pyridine at 60? C. (water bath). The solution was stirred and maintained
at the same temperature throughout the addition (30 minutes) of the solution of
2,7-fluorene[35S]disulphonylchloride, 3 g. (0.008 moles) in 60 ml. hot toluene.
The reaction mixture was stirred throughout the following 3 hours at 80? C. (a
yellow precipitate started to form after 30 minutes of stirring). The precipitate
was allowed to cool to room temperature and then collected, washed with methanol
and dried over phosphorus pentoxide. The yield of crude material was 3-5 g.
(68.7 per cent of theory), melting point 232? C. Purification from 200 ml. mixture
of N,N-dimethylformamide and dioxane (1: 1) with charcoal gave 1.9 g. (37.3 per
cent of theory) of white crystalline material melting at 296-298? C. and having a
specific activity of 730 disintegrations per second per mg. (0-02 /,c per mg.).

Analysis. Calculated for C27H16N6S208 (molecular weight 616.6) : C, 52660;
H, 2-62; N, 13-63; S, 10-40. Found: C, 52-42; H, 2*70; N, 13-49.

The presence of nitro groups in this compound was proved by their reduction
to amino groups, diazotization and coupling with /3-naphtol to give a red dye.

860

TRANSPLANTED WALKER CARCINOSARCOMA

N.N'-bis(6(2,4-dimethoxy-pyrimidyl))-2,7-fluorene[35S]disulphonamide, VII.-
2.4-Dimethoxy-6-amino-pyrimidine, 2-8 g. (0.017 moles) was mixed thoroughly
(in mortar) with 2,7-fluorene[35S]disulphonylchloride, 2 g. (0.0055 moles). The
mixture was divided into four 1-2 g. portions. Each portion was introduced into
a glass tube, 10 ml. toluene was added and tubes were sealed. Four tubes were
placed in an oil bath and temperature was raised up to 180? C. and maintained
throughout the following 10 hours. The tubes were allowed to cool to room
temperature and opened. The dark beige reaction mass which settled on the
tube walls was separated from the toluene solution, and methanol was added
(several portions) to help to remove residue from the tubes. Yield of 3-65 g. of
crude material was obtained which after drying and powdering in the mortar
was extracted with 20 ml. of toluene under refluxing. The material was dried
over phosphorus pentoxide. The yield of crude material was 3 g. (91 per cent
of theory), carbonizing within the range of 240-300? C. Purification from 20 ml.
mixture of N,N-dimethylformamide and dioxane (1: 1) with charcoal and precipi-
tation with methanol gave 1-8 g. of beige product which purified again in the same
way resulted in 0X8 g. (24-3 per cent of theory) of yellow precipitate, decomposing
at 330? C. and having a specific activity of 1330 disintegrations per second per
mg. (0X036 lic per mg.).

Analysis. Calculated for C25H24N6S208 (molecular weight 600-55): C, 49-98;
H, 4*03; N, 13.99; S, 10-86. Found: C, 49-60; H, 3.94; N, 14-12.

ANIMAL EXPERIMENTS

A total of 52 young male Wistar rats was used; 24 were employed for the
subcutaneous transplantation of Walker carcinosarcoma (which was received from
Deutsche Akademie der Wissenschaften zu Berlin, Institute fur Medizin und Bio-
logie); 28 tumour-free rats served for the control groups. A week before transplanta-
tion, the experimental animals received subcutaneous injections of hydrocortisone-
acetate suspension (15 mg. per rat in 3 doses-5 mg. each every second day).
When the tumours were 11-20 days old and weighed 3-5-20 g. (in 3 rats metastases
were noted after 17 days), the radioactive compounds were administered by tail
vein injection. Each rat was given 1-2 ml. 0-05 N NaOH containing 12 mg.
C(ompounds III, IV, V, VI or VII. Each animal was then placed in an individual
metabolism cage and killed 6 hours following administration of the compounds.
The concentration and per cent recovery of radioactive material in tissues and
excreta of rats were determined by methods described previously (Argus, Kane
and Ray, 1960; Malejka, 1965). Radioactivity measurements were made in a

Tracerlab " gas-flow GM counter of low background with automatic sample
changer. The efficiency of the instrument at 1300 volts and 35 cc per minute
GM gas flow was approximately 15 per cent.

RESULTS AND DISCUSSION

The complete tissue distribution of five new fluorene disulphonamides labelled
with sulphur-35 (Compounds III, IV, V, VI and VII) in Wistar rats bearing a
transplanted Walker carcinosarcoma and tumour-free animals was compared.
This investigation was conducted with a 6 hour time interval between administra-
tion of the compounds and killing the animals in order to obtain data which

861

862                              DANUTA MALEJKA

TABLE 1-A.-Distribution of Radioactivity in Tumour-Bearing and Tumour-Free

Wistar Rats 6 Hours After Intravenous Injection of Fluorene, Disulphonamides
Labelled with Sulphur-35.*

N,N'-bis(carbamyl)-2,7-fluorene  N,N'-bis(p-tolylsulphonyl-carhI)a-

[335S]disulphonamide, III    myl-n-butyl)-2,7-fluorene[35S]

disulphonamide, IV

Experimental      Control       Experimental      Control

Groupt         Groupt          Groupt          Groupt

Per            Per              Per            Per
Tissue         ug./g.  cent    /Lg./g.  cent   lig./g.  cent  uIg. /g.  cent
Blood Cells?               0    0 00      0    (000       0    0 00       1$  0C 04
Blood Plasma?              2    0.05      0** 0*00        5    015      251,  0 * 73
Brain                        0 000        0    000       0    0-00       0   0-(4
Liver            .         7    0-33      0    000        3    0-13       8    0-28
Lungs                      0    000       0    0 00       4    0-02      11    01- -
Spleen               .     8   004        0    0000       7    0-03       5   4)-02
Kidneys     .   .    .    15    015       9    010   .    7    0-05      30   0-43
Skin   .    .   .    .    32+  4-61       8    1-80      2 4   4-84      65   10-98
Leg muscle  .         .  .  a   015       0    0 00  .    0    0-00       2    0-02
Stomach-+-contents.  .     7    0-07     11    0-15  .    10  0(06        8   4)11
Small intestine +contents  331l  2-81    26    1-37      58    2-33      35    1-40
Large intestine+contents  174   7-23    158    6-88  .   349   8-39     212    5-40
Carcass     .   .    .     0    0 00      0    0 00  .    8    3-41       8    3-73
Urine? .    .    .   . 8642    80-61  10717   91-83  . 8600   71-68    8080   67-36
Faeces .    .   .    .   862?   0-85     77    0-16  .  no              137** 0-21

faeces

Tumour      .   .    .     0    000         -        .    10  044

Total  .    .   .    .         96-90         102-29  .        91-33          9(0 86

* Dose of Compound III, IV, V, VI or VII 12 mg. per rat.
t Average value from 6 rats.
+ Average value from 4 rats.

? Concentration in Mg. compound per ml.; other data are in pg. compound per g. tissue.

Average value from 5 rats.
Average value from 2 rats.
** Average value from 3 rats.

could be compared to those for Compounds I and II (Malejka, 1965), and for
other fluorene disulphonamides tested previously (Argus, Kane and Ray, 1960:
Malejka, Argus and Ray, 1961 ; Malejka, 1962). The details of present studies
for Compounds III-VII are shown in Tables I-A and I-B; concentrations are
expressed in 4ag. of compound per g. tissue or ml. blood or urine, and in percentages
of administered dose recovered.

Substituents   in  the  2,7-fluorene-disulphonamido    molecule   influence  the
physico-chemical properties and biological behaviour of the resulting compoulnds.
The chemical structure of N,N'-bis(carbamyl)-2,7-fluorene[35S]disulphonamide.
III, with the lowest molecular weight (410.3) of Compounds I VII, and its easy
solubility in water relate Compound III to the bis-guanidyl derivative, II. tested
previously (Malejka, 1965). Six hours after administration, Compound III is
almost completely excreted (trace amounts of radioactivity remain in the kidnev)
into urine in which in experimental group-80-61 per cent, and in controls- 91-83
per cent of the given dose is recovered: the difference between the two groups
is statistically significant at P - 0 05 (Table II). This finding together with the

TRANSPLANTED WALKER CARCINOSARCOMA       863

0~~~~~~~~~

0 0.~                  1
0  I.,

0  o~~u

bo -

.EE I-"   XO00>>0ss< Q          1

-  C~~~~~~~~~~C

@   ;    I  -     E O C 00 E r- o _ 00C rt   O  k__
Z            d4   to 001  01 r-ooo_u  0e o  0

ct~    .o    .  *. . .-      _

-) ^Y E e  X  u0000-WZ000co 0W 00

0~~~~~~~~~~~~~~0

Q e tO  E =  0-0 00  00 -O 4N0

00

0     0                              0 I
u  <  >, 4;)   ; ;  ce ce oola  t- o o*  t- laz t- u   co D_ o s c   e

*W  :~ .s at I ,  ;4 8 .?>?.  --occs,t - oo -

az~~~~~~~~~~~~~~~~~ xm Itz? .

~~~~~~~~~~~~~a        ._   Vt _cr_00c0_o

PZ41     L

",,  4; .a  0_          e n s O

~~~ ~~~>  r  ~~~~~~~~        0 ~0 >

CO Z  I"v 5   0-40lO   0   00>

~~  0~~  -  -~~       .Hc 0r Zc,

I         P4   o    0 C  to km0

L                         -

-4not~  I 0                      X 0

t      S* Is    55 oooo_c5ono 0-

0   C
.X o  *.ok?

O             0

as  (04  744

a      r  r  ;  ;  0 < "~~~~~~~~~~~~~~~~~+  >  _>  > >  *.

I.        0    .~~~~~~~~~~-4 ...  00    o  0-

bi~~~~~~~~~~~~~~~~~~~~~~~~.   a   4-  as   O  Ca  S   ==

+ o          0 0 v  0  0 0

*y~~~~~~~~~~~~~~~~~~~~~~..       > > 0.

0 00
0 0

P    4 ie  ;4I

*  *  - -4 < 4  t4  Oo

H        ;        -C  a  te ) oWOOOh0~00  ?-~  * M  1-gOOOeOOOaoNc

DANUTA MALEJKA

10       10*   -

e ++    ao * CO

->     0 0 al  01~ c C'.l10

0        041   -0

I      aq       N    P1+

0

0m

;>      4       I o, P. s

m           V -

-  41

~~     I o~~C

0
H   00  V CX m

00

+   1CV 10C~".4  10

O V'

- H
O

01

0     0

m   V10 -H

'14      C;I*

00
01

C;

4+
-   I-  01"? -

H   j >   E O  O H8

0VW

0e

01~~~~,1

O

e  *   -01* -

0

0.

.~~

o v

-N " V 3DH

O 10
0

++

OC>

4 ~V-

I-

o
10~ ~~e

V

00
**V. * -

OO

-o   vo   t -
+10  10

C>  0 no +

10

0 0

_q  o

00
0o

t--

00  0

10  00 1

0~~~~~0
10

0i

0

0

L 10

0

0o

O

In
t-
Cr;

V I

r0     0

*  -H  0
I0 eOO 1,
?o  km to

C     O10

10   0 1

864

0

0

.C

14.4 14.4

* *     *

Ca 7  -s  - Cs:

b to

3 F=*

*-4

o6

bO

0

1~4
0

0

0

0

0*
Co~

co0
*C.-a

02

,4 I
Z s
M.Q

4 - j
Zt

0eb

.

ii

H*ct

TRANSPLANTED WALKER CARCINOSARCOMA

fact that some radioactivity remained in organs of tumour-bearing rats indicate
an impaired elimination of the 35S-labelled compound in the presence of tumour.
In general, the distribution of Compound III occurs very similarly to the bis-
guanidyl derivative (Compound II). Among other factors, no radioactivitv of
Compound III is found in tumour which suggests a lack of affinity of the bis-
carbamyl derivative for protein binding.

Compound III forms a basic structure for Compound IV-N,N'-bis(p-tolyl-
sulphonyl-carbamyl-n-butyl)-2,7-fluorene[35S]disulphonamide-in which hydro-
gens of both urea nitrogens are symmetrically substituted with n-butyl- and
p-tolylsulphonyl-groups. A " double " bis-disulphonamide has thus been obtained
with the highest molecular weight (835) of all fluorene disulphonamide derivatives
tested. Despite the high molecular weight of Compound IV, polar nature of the
substituents added contributes to its solubility in water. Hence, only differences
which might be due to its high molecular weight are noted in the distribution of
Compound IV compared to the bis-carbamyl derivative, III. Compound IV is
excreted into urine at a slower rate than Compound III: 71-68 per cent of the
given dose is obtained in the experimental group, and 67-36 per cent in the con-
trols; this is not a significant difference. As the result of the lower values for
urine, more Compound IV than III is found in the other tissues and organs of
rats. The differences, however, in the distribution of Compound IV between
tumour-free and tumour-bearing rats are not statistically significant (Table II).
In tumour, 10 pg. of compound per g. tissue is found; this will be discussed
later.

The behaviour of N,N'-bis(4-carboxy-phenyl)-2,7-fluorene[35S]disulphonamide,
V, may be compared with the disulphonamides known from the earlier studies.
In going from the biphenyl to the fluorene series, the activities of the compounds
are not parallel. In the biphenyl series, compound with free carboxylic groups
examined in CAF1/Jax mice with a transplanted stomach carcinomata (2 and 8
hours after its administration) showed more selective localization in tumour than
the derivative with primary sulphonamido groups (Argus, Seepe, Gutierrez,
Hewson, and Ray, 1958). In the fluorene series, the effect was the opposite:
the comparison of Compound V with N,N'-bis(p-sulphamoyl-phenyl)-2,7-fluorene
[35S]disulphonamide (Malejka, 1962) in Wistar rats bearing Walker carcino-
sarcoma has not proved a specific affinity of the free-carboxylic groups occurring
in Compound V to tumour cells. Six hours after administration, N,N'-bis(4-
carboxy-phenyl)-2,7-fluorene[35S]disulphonamide, V, is excreted mainly through
the gastrointestinal tract. Considerable amounts of radioactivity are found in
small and large intestines-13-57 and 65-52 per cent of the given dose in the
experimental group and 10-47 and 60-33 per cent of the dose in the controls,
respectively (Table I-B). The lower values for the control group are mostly
compensated by the activity found in faeces-6*08 per cent of the dose given.
In the tumour-bearers radioactivity is not found in faeces, and about j- less
compound is found in urine of this group than in tumour-free animals (Table II),
thus showing similarity to the distribution of Compound III.

N,N'-bis(5(6)-nitro-benzimidazolyl)-2,7-fluorene[35S]disulphonamide, VI, was
obtained in the reaction of 2,7-fluorene[35S]disulphonylchloride with imino-
nitrogen of 5(6)-nitro-benzimidazole. The nitro groups at C5 or C6 of benzene
ring as electronegative substituents were expected to increase tumour localization
of the compound. Data obtained for the distribution of Compound VI in tumour-

865

DANUTA MALEJKA

bearing and tumour-free Wistar rats show its very similar behaviour to Com-
pound IV (Tables I-A, I-B and Table II). The excretion of Compound VI into
urine remains on a similar level to Compound IV; the concentration of radio-
activity in tumour was very much the same.

A portion of the sulphadimethoxine molecule, a sulphonamide known to
remain at elevated blood levels for prolonged periods of time, appears in N,N'-bis
(6(2,4-dimethoxy-pyrimidyl))-2,7-fluorene[35S]disulphonamide, VII, which simi-
larly to the bis-thiazole derivative, I, (Malejka, 1965) is insoluble in water and
requires an alkaline pH to dissolve. Compound VII has a higher molecular
weight (600.5) than Compound I (490.6). These physico-chemical properties
might account for the slow elimination of Compound VII in the rat, and this is
slower in the experimental animals than in the controls. In the skin of tumour
bearing animals twice as much, and in the small intestines (+ contents) 3 times
more, of Compound VII is found as in the controls. (The difference between the
two groups of animals for small intestines is at P < 0*001 (t = 7.70). On the
other hand, in the large intestines (-+ contents) of tumour-free animals 35 times
more and in the urine 2 times more of Compound VII is present than in the
tumour-bearing group. The differences in the concentration of Compound VII
between the two groups found in the large intestines and in the urine are statisti-
cally significant (t = 8X82, P < 0-001 and P _ 0 05 in Table II, respectively).

The response of the reticulo-endothelial system towards the uptake of fluorene
disulphonamides is different for every compound presented in this paper. Com-
pound III deviates from the behaviour of four other disulphonamides in that some
radioactivity is found in the liver and spleen of tumour-bearers but not in these
organs of the control group. This may indicate that only liver impaired by the
presence of a tumour in the animal body can take up the compound, although the
effect is not statistically significant. Compounds IV, V, and VI localize to a
greater extent in the liver of tumour-free animals than in the liver of the tumour-
bearers (ratios 2X67, 2.57 and oo, respectively, in Table II). The difference in the
uptake of the compounds by the control and experimental liver is statistically
significant only for Compound VI (0.02 < P < 0.05). The content of Compound
VII in the liver of both groups of animals is similar (ratio 0.93). In the spleen of
tumour-free rats 1-5 times more Compound VII is found than in the presence of
tumour (0.05 < P < 0.1), and also larger amounts of Compounds V and VI are
found in this organ of the control group than in the tumour-bearers (the differences
between the two groups for Compounds V and VI are not, however, significant).
Thus, in general, the four disulphonamides (Compounds IV, V, VI and VII)
tend to follow the behaviour of the pattern compound: N,N'-bis(naphthalene)-
2,7-fluorene[35S]disulphonamide with which an impaired function of the reticulo-
endothelial system in the presence of tumour has been shown (Argus, 1961;
Argus, Hudson, Seepe, Kane and Ray, 1962).

The aspect of a possible diagnostic value of recently obtained disulphonamides
can be discussed only in relation to Compounds IV, VI and VII for which radio-
activity is found in tumour 6 hours after their intravenous injections into experi-
mental rats. In Table III the ratios of the concentration of radioactivity in
tumour to the concentration in other tissues for the three disulphonamides and
also for Compound I (Malejka, 1965) are presented.

As has been pointed out, the distributions of Compounds IV and VI appear to
be very similar as are also the values of their concentrations in tumour. For

866

TRANSPLANTED WALKER CARCINOSARCOMA

K :

MQ-

eb ~
1-4 o

H
O-4

0
0 4
0

-

04

S

0
04

0

0

01
_   0

So Vo

O O \ /

v II I

01
;~~~~C

-4

o <I

zIIV

.0  .   .  .  .

o

*

?o 0

o      I0 0

0.. . .

0

0   = 0   - 4  10o -   0 1 0

Co C0 0C   C0   CO

p  ;  0 0 0 I   01 0 O

00000000

CO4  P-4   P01 0 1 PCO

P0

vI  zi  i  I zizi   II
o  - _o co c> o

t;> o  reo co

0

01  ~ ~ m00

4+

0  0 1-- .  -- C *   - C)

q  1 4 0 4 0 C - 4

0       0   CC  0

00

C1)

0.4
C)
C1)

0

C)
0

Cs
0

C)C)5      .4

00"M

0     0 04  ) 5

H -

0-4

0
C)
CD

CI

C)

0

14
9

.

C)

0

0

04-
00

C)

-4

.

a)
0.

..a
I
0

0
C)

0*

867

0
0

Cs
14-

C)

4.

0

>
C)

0.

?)
0

* -

?   0

00 0

14 4
-C

H~ 00

-Y

oo0
fZ C
0   00

-~4p

i

I

DANUTA MALEJKA

Compound IV all ratios of the concentration in tumour to the concentrations in
blood cells and plasma, liver, lungs, spleen, kidneys, leg muscle and carcass were
greater than 1O00. The ratios obtained for blood cells and leg muscle (20.50)
are of a great statistical significance (P < 0.001), also ratios for liver (5.40) and
lungs (2.71) are significant (P - 0.05). The most favourable ratios for Compound
VI from the statistical point of view are those obtained for liver (19.00 at
P < 0.001),redbloodcells (16-21 atP _ 0-02)andlungs (13.04at0 05 < P < 0 1)
The favourable ratios obtained for the other tissues for Compound IV. and for
leg muscle and carcass for Compound VI are not of statistical significance; however,
this does not exclude that a significant difference may be found, using larger
numbers of animals.

The behaviour of Compound VII in Wistar rats can be compared to the bis-
thiazole derivative (Compound I) (Malejka, 1965). It has been shown bv means
of radioelectrophoresis that the latter compound is strongly attached to rat and
human serum albumins (Malejka, Kasprzak and Radola, 1966). This protein-
binding ability might account for a high concentration of Compound I in the liver
and spleen and also in tumour. The ratios of the concentration of Compound I
in tumour to its concentration in blood cells, leg muscle and carcass exceeded
1.00 (1.50 at P- 005; 3-18 at 0.001 <P < 0.01 ; 9*43 at P_ 001. respec-
tively) ; the ratios for plasma, liver, lung, spleen and kidneys are smaller than
1.00.

The blood level of Compound VII (Table I-B) 6 hours after administration is
not as high as Compound I (Malejka, 1965). Despite the less persistent plasma
protein-binding ability of Compound VII, a more selective concentration in
tumour is achieved with this derivative than with Compound I, as shown by the
ratios in Table III for red blood cells (8.50 at P < 0.001). blood plasma (1.89 at
P-- 0-05), lungs (1.57 at 041 < P < 0.2), leg muscle (10.9 at P - 0.02) and
carcass (2-65 at P < 0.001). The ratios of the concentration of Compound VII
in tumour to the concentration in liver, spleen and kidneys, however. remain
smaller than 100. Compound VII, nevertheless, presents a most promising
structure in searching for favourable localization in tumour, and encourages a
further investigation of sulphonamido derivatives with heterocyclic rings.

CONCLUSIONS

1. The fluorene disulphonamides with aliphatic side chains symmetrically
attached to 2,7-fluorene molecule, N,N'-bis(guanidyl)-2,7-fluorene[35S]disulphona-
mide, II, and N,N'-bis(carbamyl)-2,7-fluorene[35S]disulphonamide, III, result in
rapid elimination from rat and show no tendency to localize in tumour.

2. Two high molecular weight disulphonamides: N,N'-bis(p-tolylsulphonyl-
carbamyl-n-butyl)-2,7-fluorene[35S]disulphonamide, IV, and N,N'-bis(5(6)-nitro-
benzimidazolyl)-2,7-fluorene[35S]disulphonamide, VI, with electronegative substi-
tuents in their molecules (sulphono-groups in IV and nitro-groups in VI) give
similar distribution data and very similar concentration in tumour. The ratios
of the concentration in tumour to the concentration in red blood cells. liver,
lungs and also in plasma, leg muscle and carcass are favourable.

3. N,N'-bis(4-carboxy-phenyl)-2,7-fluorene[35S]disulphonamide, V, despite its
free carboxylic groups is not found in tumour.

86S

TRANSPLANTED WALKER CARCINOSARCOMA

4. The compounds with heterocyclic rings: N,N'-bis(thiazole)-2,7-fluorene
135S]disulphonamide, I, and N,N'-bis(6(2,4-dimethoxy-pyrimidyl))-2,7-fluorene
[35S]disulphonamide.. VII, reveal appreciable concentrations in tumour (35 ,ug.
and 77 lig. per g. tissue, respectively). It seems, however, that a strong protein-
binding ability of Compound I passed its maximum for a diagnostic effect (ratios
of the concentration of Compound I in tumour to the concentrations in plasma,
liver, lungs, spleen and kidneys are not favourable) while a protein-binding level
reached with Compound VII exhibits its more selective concentration in tumour
(ratios for red blood cells. plasma, lungs, leg muscle and carcass are favourable).

SUMMARY

Five new radioactive compounds: N,N'-bis(carbamyl)-2,7-fluorene[35S]disul-
phonamide, III, N,N'-bis(p-tolylsulphonyl-carbamyl-n-butyl)-2,7-fluorene[35S]di-
sulphonamide, IV, N,N'-bis(4-carboxy-phenyl)-2,7-fluorene[35S]disulphonamide,
v. N.N'-bis(5(6)-nitro-benzimidazolyl)-2,7-fluorene[35S]disulphonamide, VI, and
N,N'-bis(6(2,4-dimethoxypyrimidyl))-2, 7-fluorene[35S]disulphonamide, VII, were
synthesized, and their tissue distribution 6 hours after a single intravenous injec-
tion to tumour-bearing (Walker carcinosarcoma) and tumour-free Wistar rats
was studied. The data obtained are discussed in relation to the bis-thiazole
(Compound I) and bis-guanidyl (Compound II) derivatives examined previously
(Malejka. 1965). This similarity of chemical structure of the substituents at
2,7-disulphonamido-fluorene draws a line of common factors in the behaviour of
Compounds II and III, Compounds IV and VI, and Compounds I and VII. The
distribution data for these paired compounds are similar. Compounds II and III
are not present in tumour. The concentration in tumour of Compounds IV and
VTI is very similar and reveals favourable ratios for red blood cells, liver, lungs
(statistically significant), and for plasma, leg muscle and carcass for both com-
pounds. and also for spleen and kidneys for Compound IV. The highest concen-
tration in tumour (77 f,g. per g. tissue) is achieved with Compound VII and this
value gives favourable ratios for red blood cells, plasma, lungs, leg muscle and
carcass (all statistically significant).

The present studies have shown an impaired elimination of fluorene disulphona-
mides in presence of tumour with Compounds III, V and VII. Four of the
examined compounds (IV, V, VI and VII) tend to localize in the liver and spleen
of tumour-free rats to a greater extent than in tumour-bearers, thus showing a
weakened phagocytic function of the reticulo-endothelial system in the presence
of tumour.

The author is very grateful to the Professors Francis E. Ray and Mary F.
Argus for their valuable comments on the manuscript. The author is very much
indebted to Professor Ewaryst Pawelczyk for his encouragement in conducting
these studies. The author wishes to thank Professor Przemyslaw Gabryel and
Mr. Kazimierz Kasprzak for their kind hospitality at the Isotropic Laboratory
of the Department of Pathological Anatomy. The use of " Tracerlab " equipment
at the Isotopic Center of the IlIrd Intern Clinic of Poznan Medical Academy is
gratefully acknowledged.

869

870                     DANUTA MALEJKA

REFERENCES

ARGUS, M. F.-(1961) Bull. Tulane med. Fac., 20, 151.

ARGuS, M. F. AND HEWSON, K.-(1954) Br. J. Cancer, 8, 698.

ARGUS, M. F., HUDSON, M. T., SEEPE, T. L., KANE, J. F. AND RAY, F. E.-(1962?

Br. J. Cancer, 16, 494.

ARGUS, M. F., KANE, J. F. AND RAY, F. E.-(1960) Proc. Soc. exp. Biol. Med., 103, 87.
ARGUS, M. F., SEEPE, T. L., GulERREZ, N., HEWSON, K. AND RAY, F. E.-(1958)

Br. J. Cancer, 12, 636.

KASPRZAK, K., MALEJKA, D. AND GABRYEL, P.-(1965) Nature, Lond., 206, 1266.
MALEJKA, D.-(1962) Br. J. Cancer, 16, 170.-(1965) Br. J. Cancer, 19, 513.
MALEJKA, D., ARGUS, M. F. AND RAY, F. E.-(1961) Cancer Re8., 21, 673.

MALEJKA, D., KASPRZAK, K., AND RADOLA, P.-(1966) Patol. pol. (in press).

				


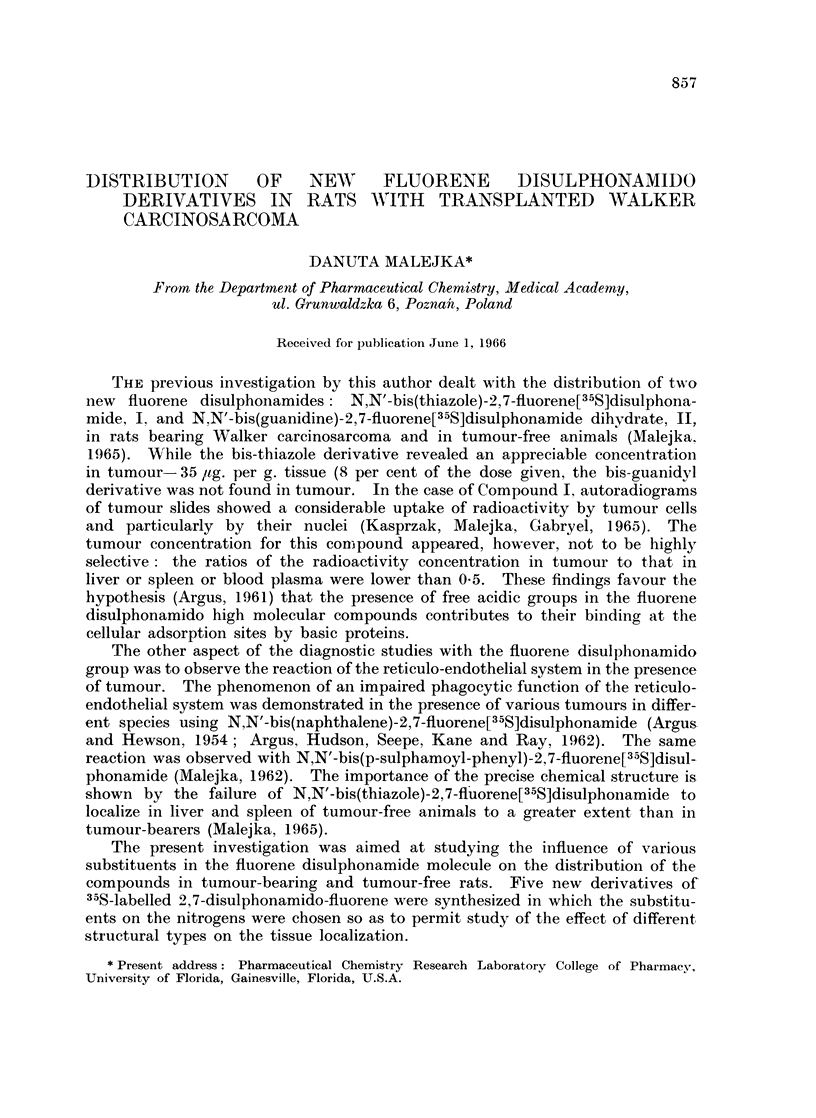

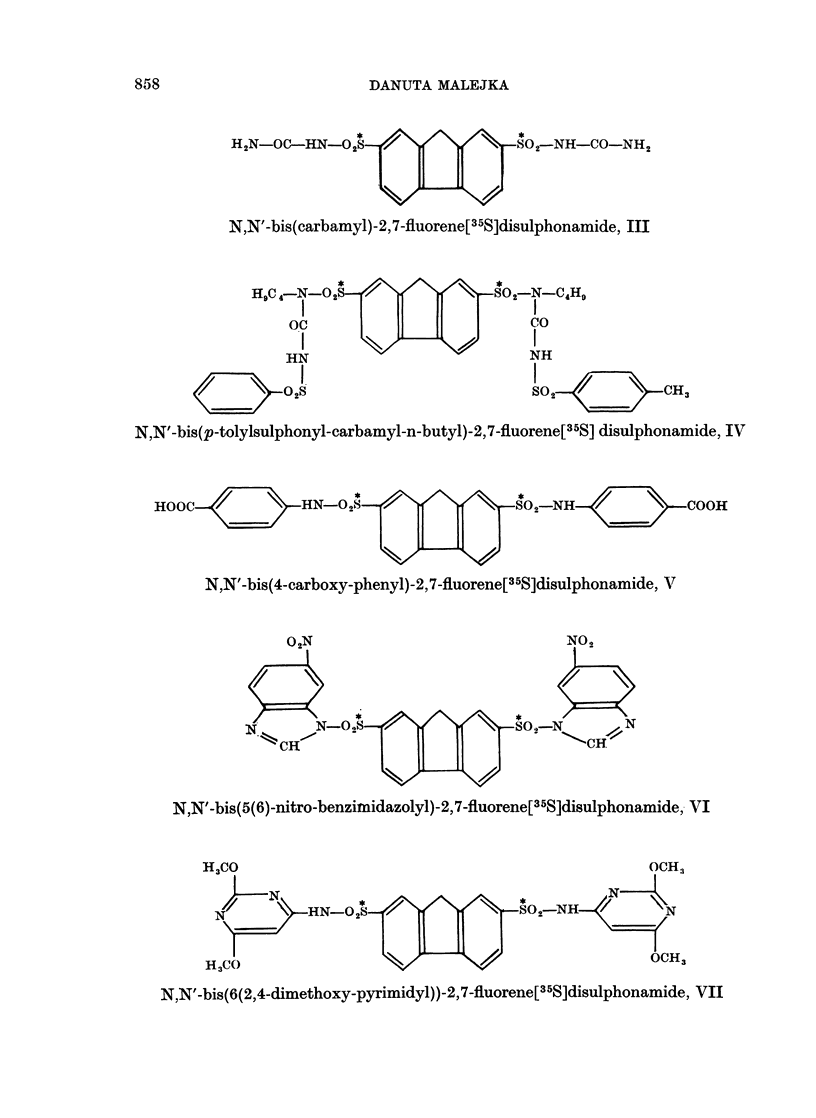

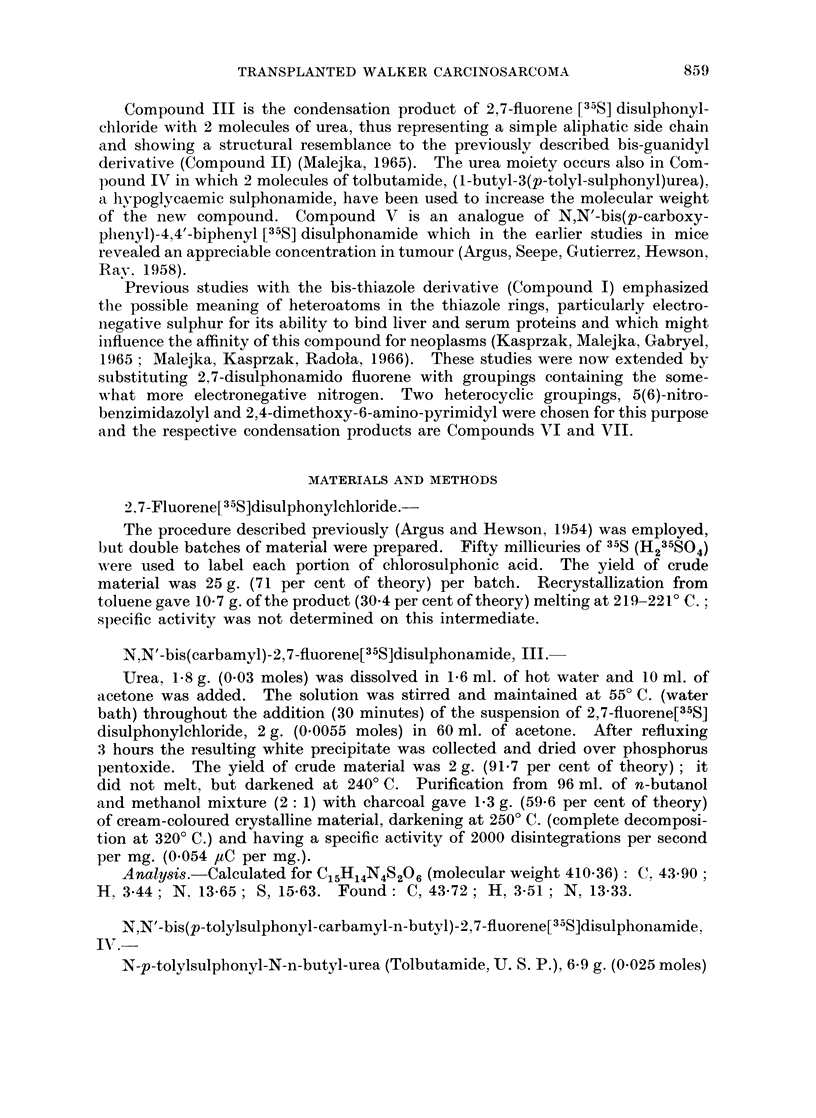

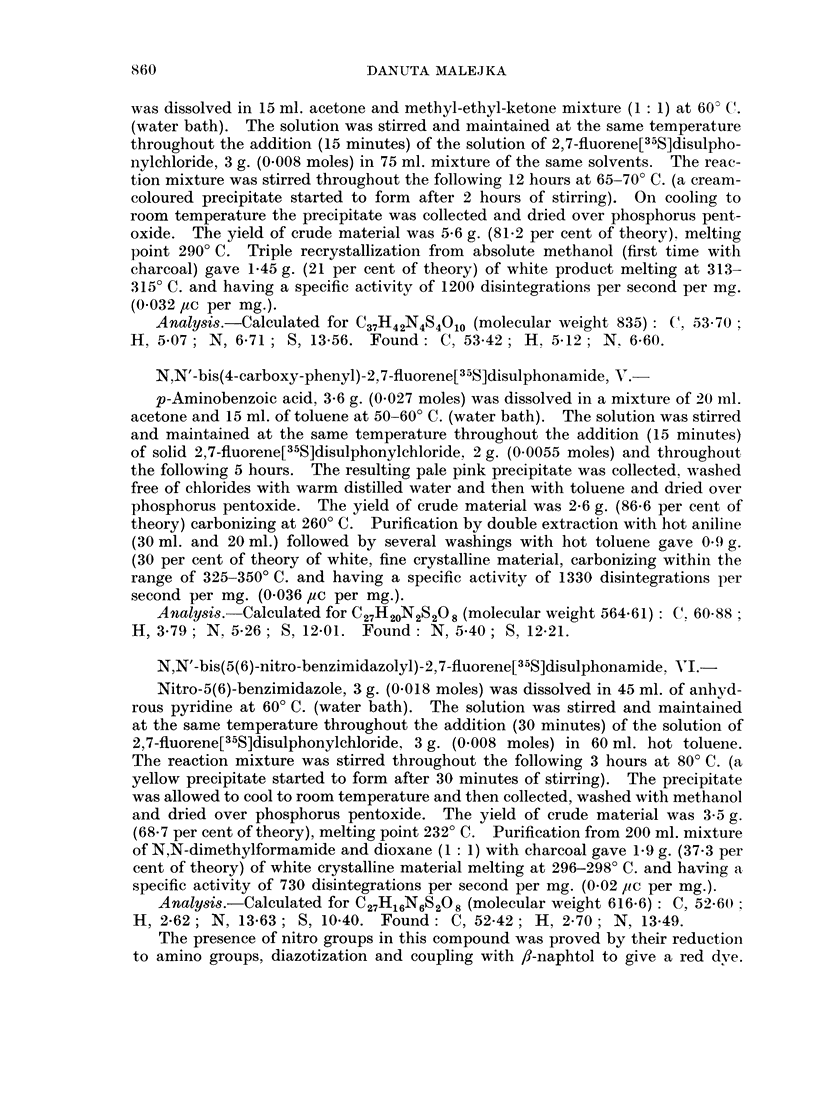

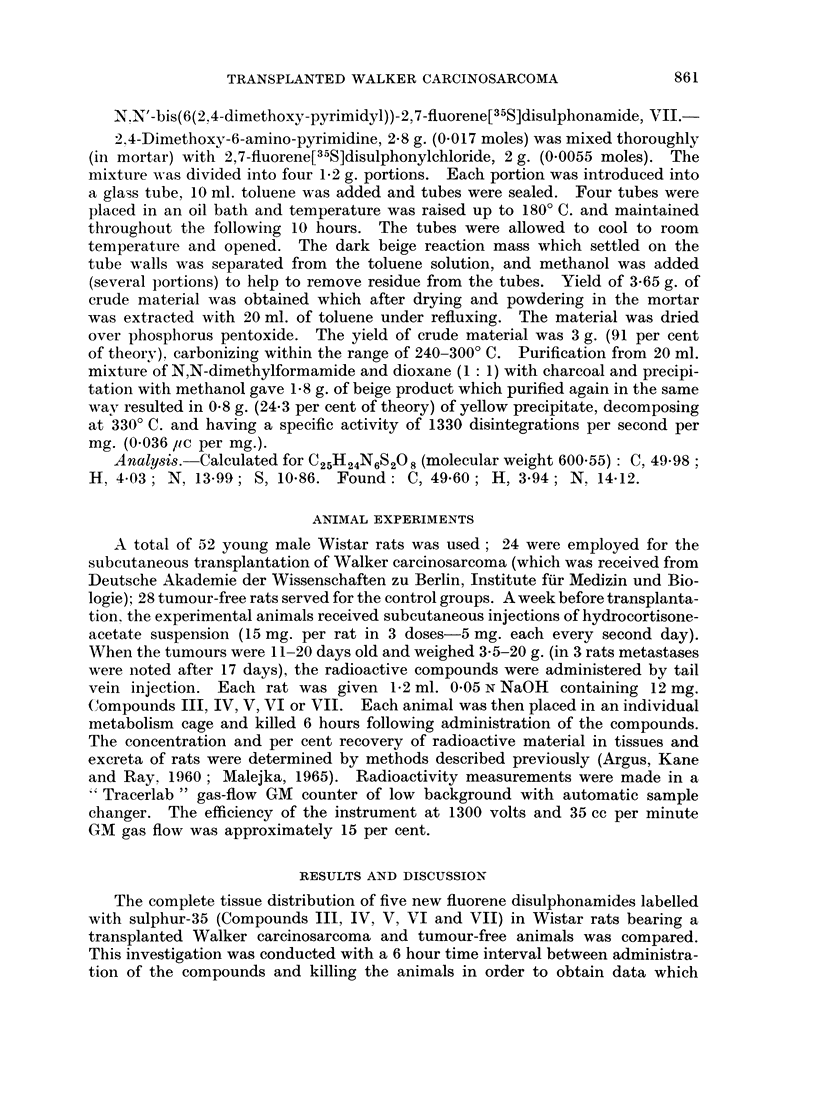

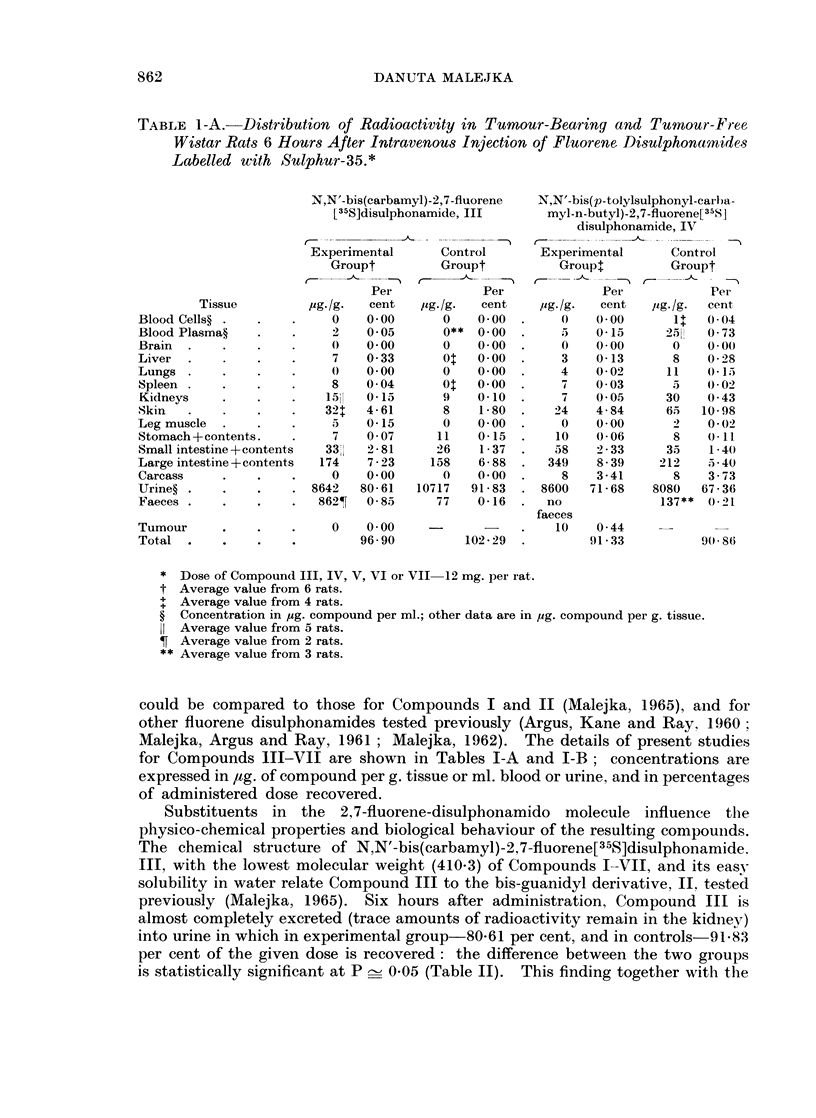

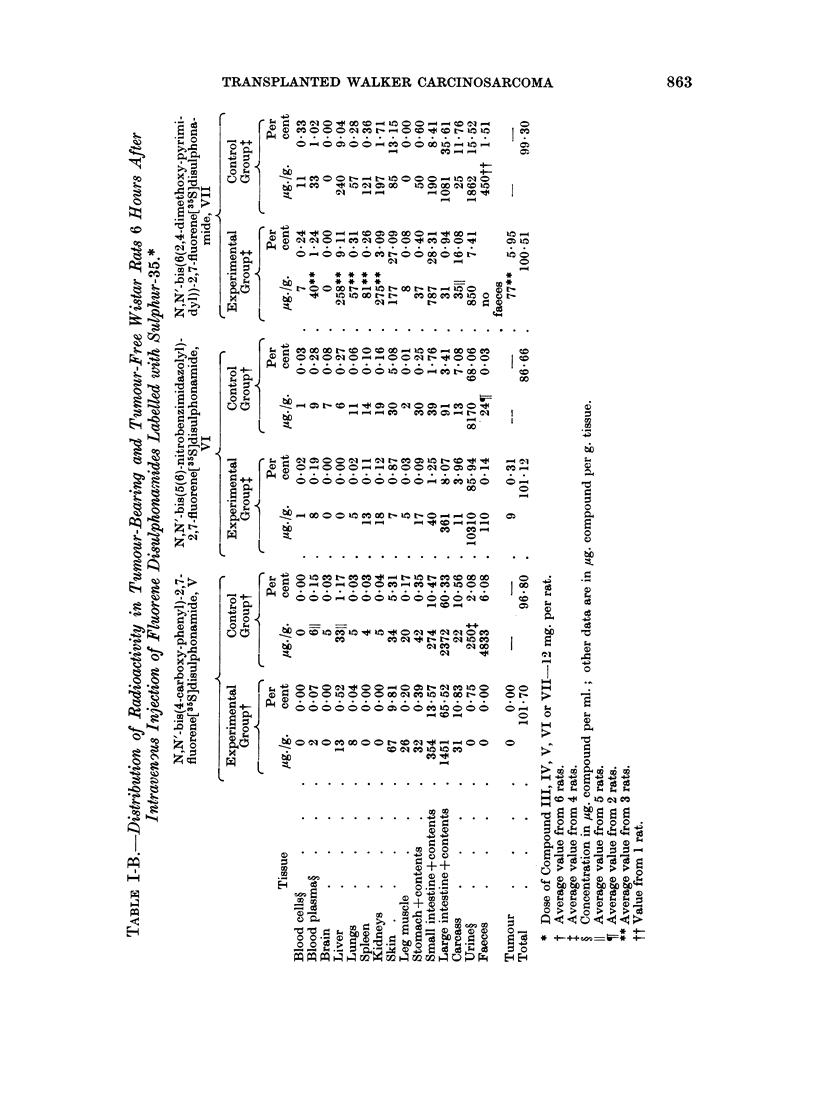

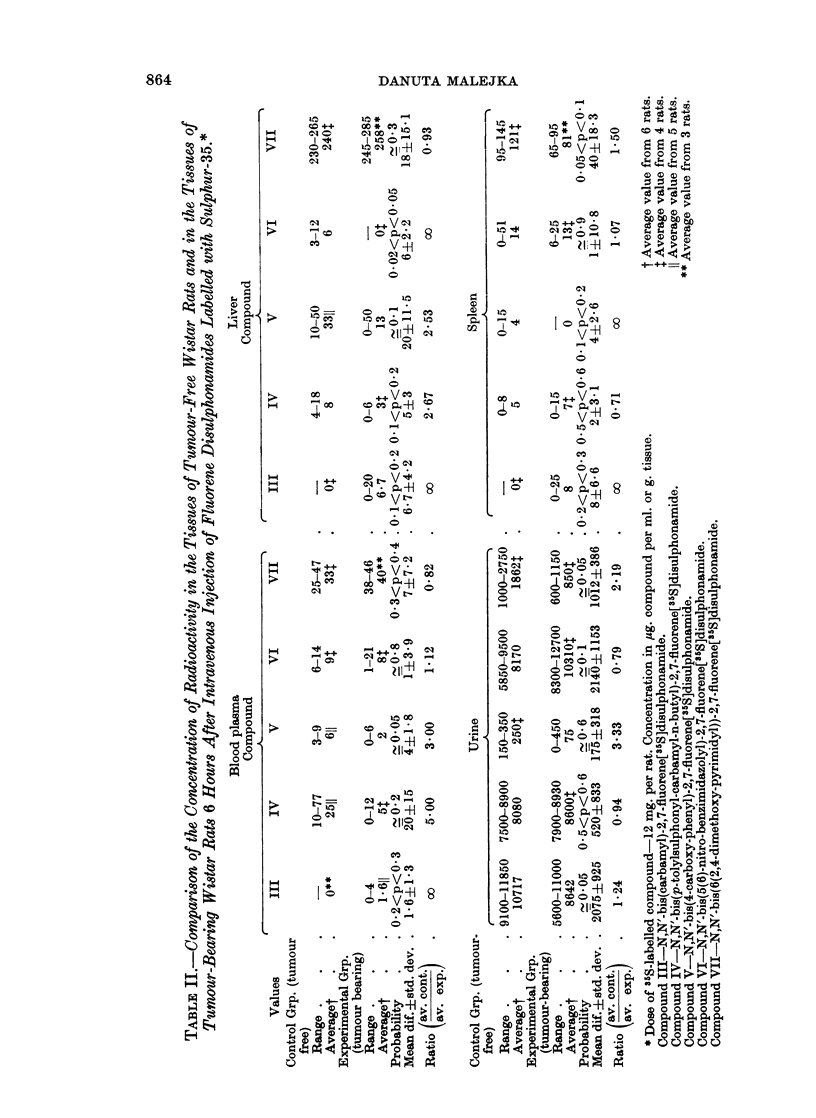

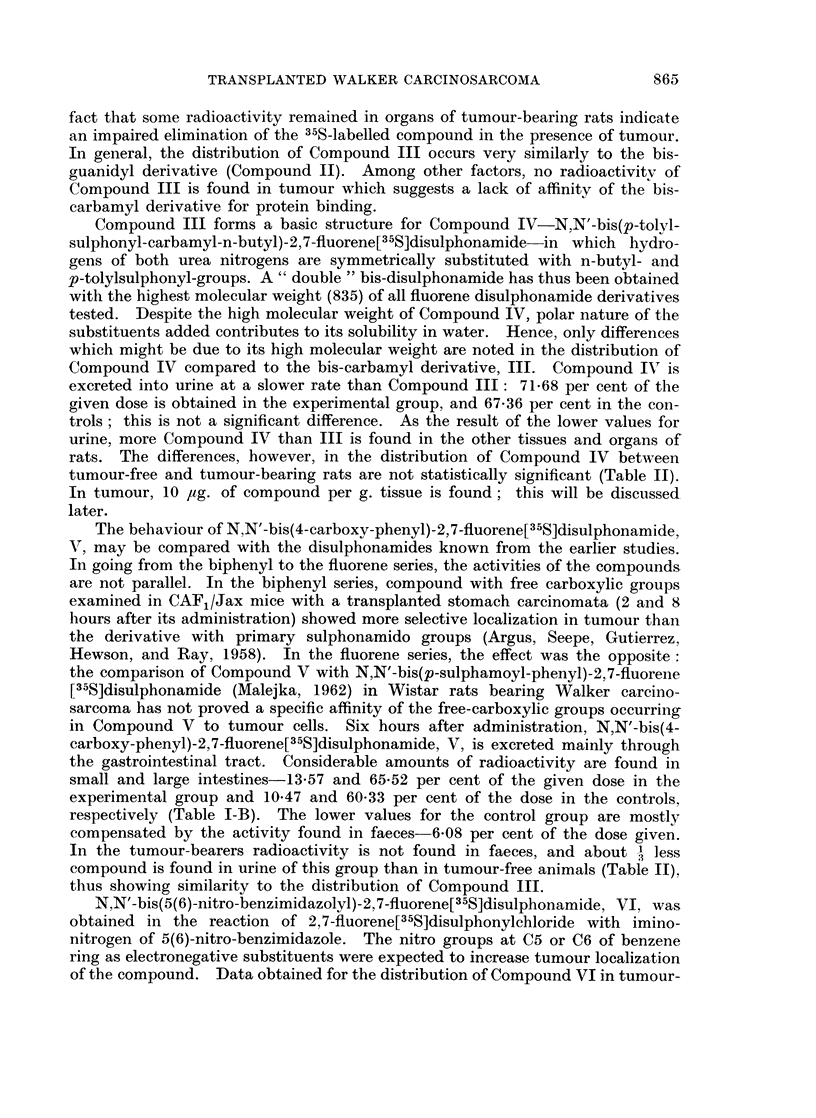

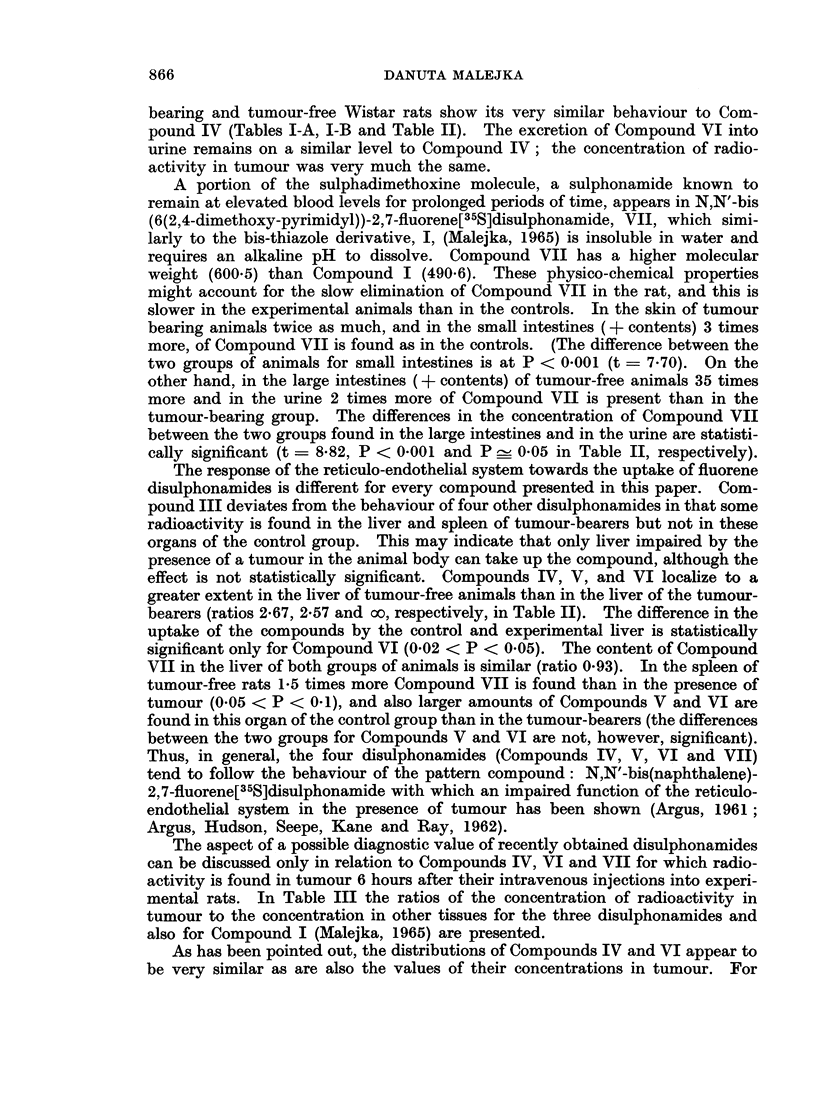

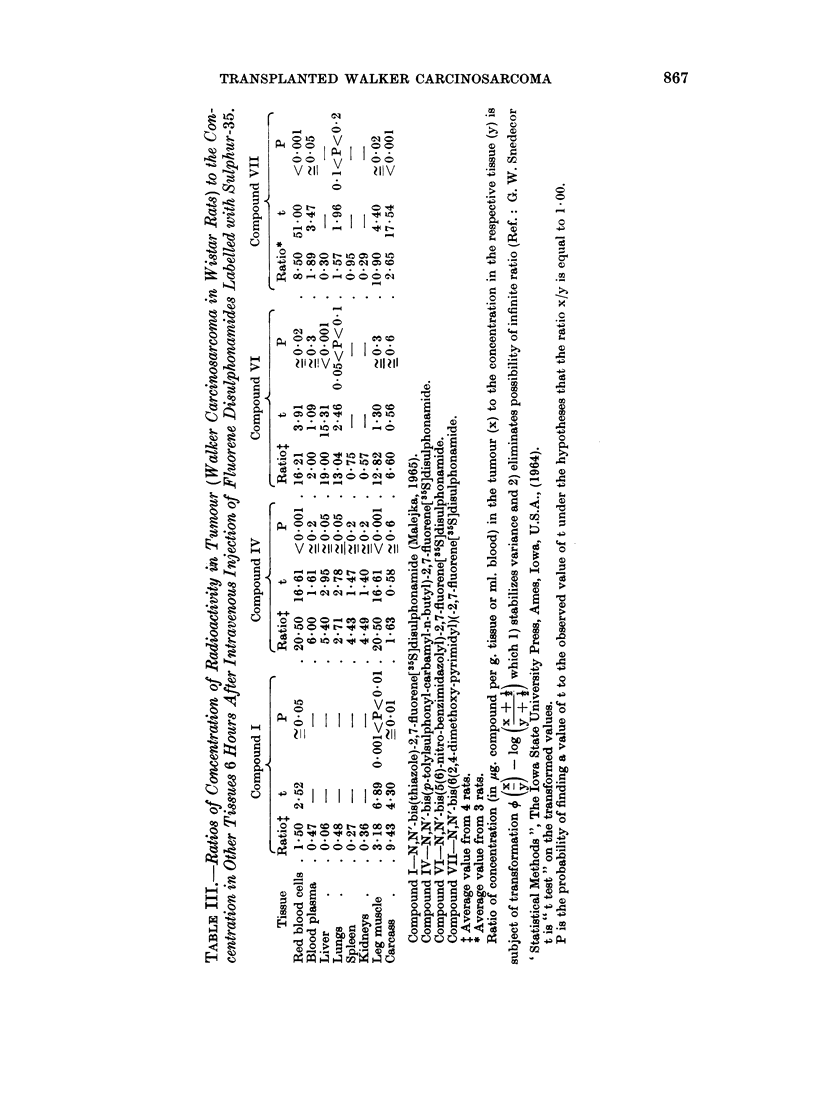

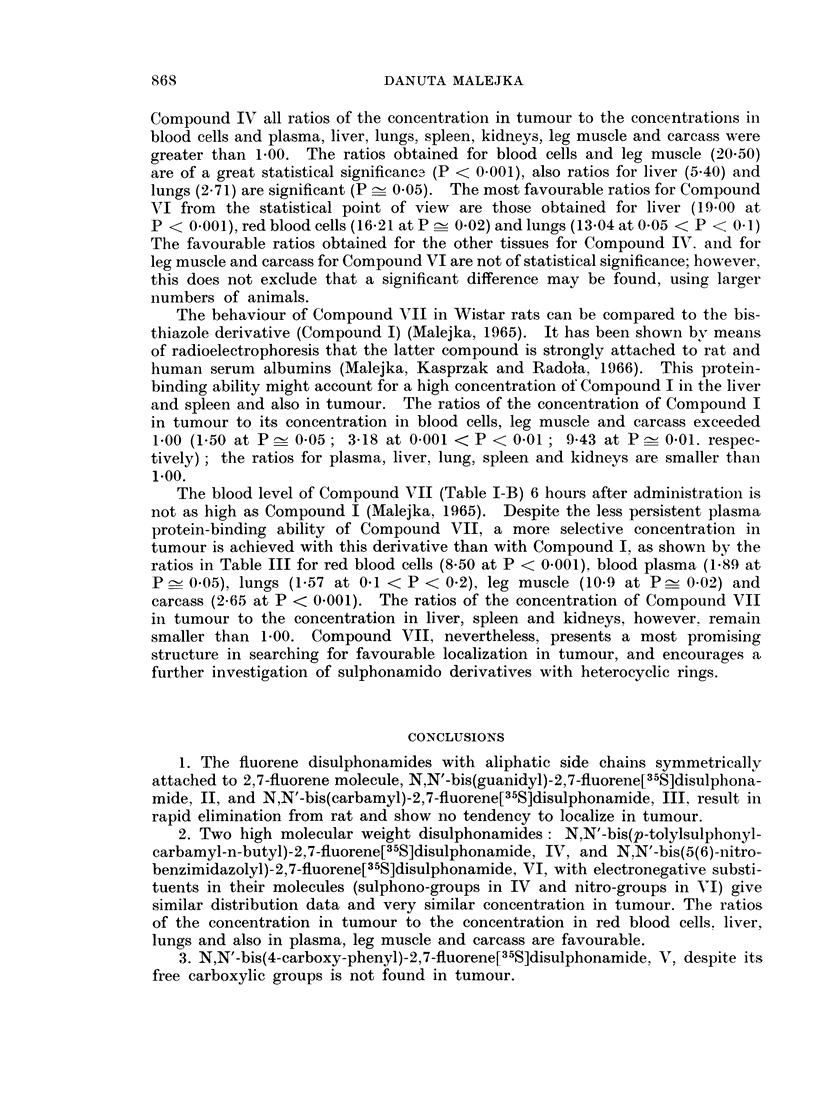

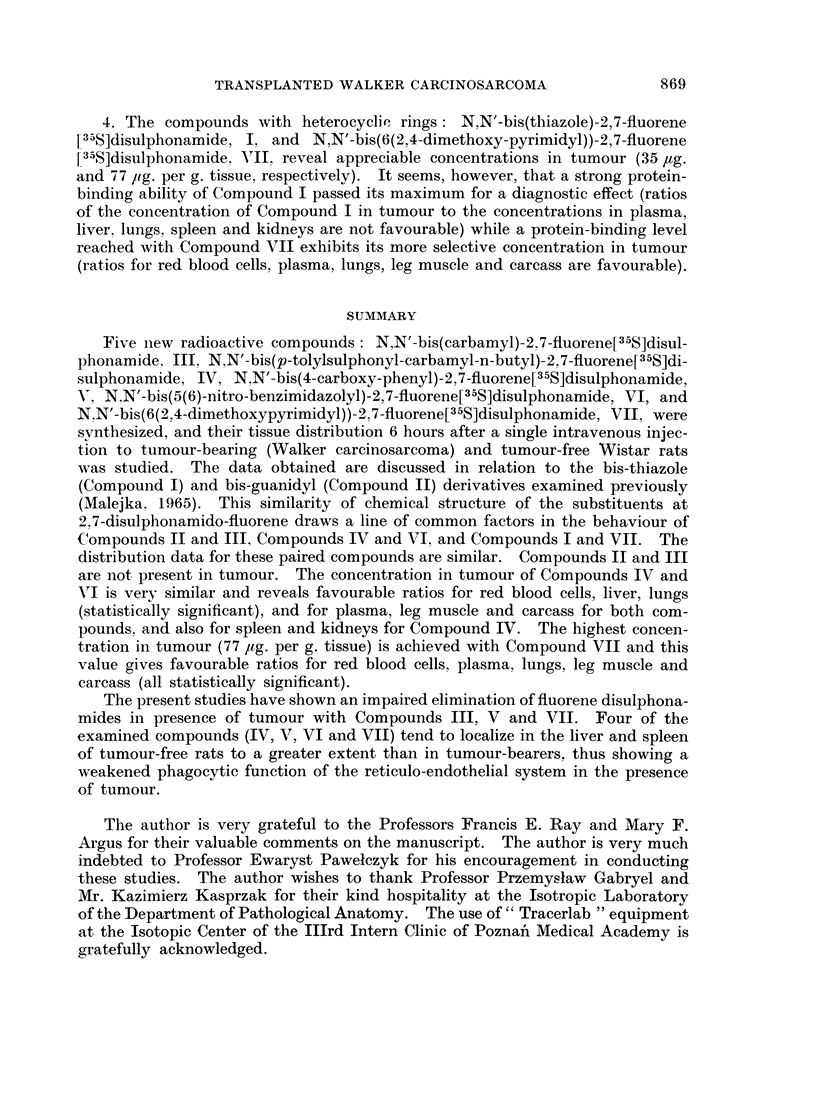

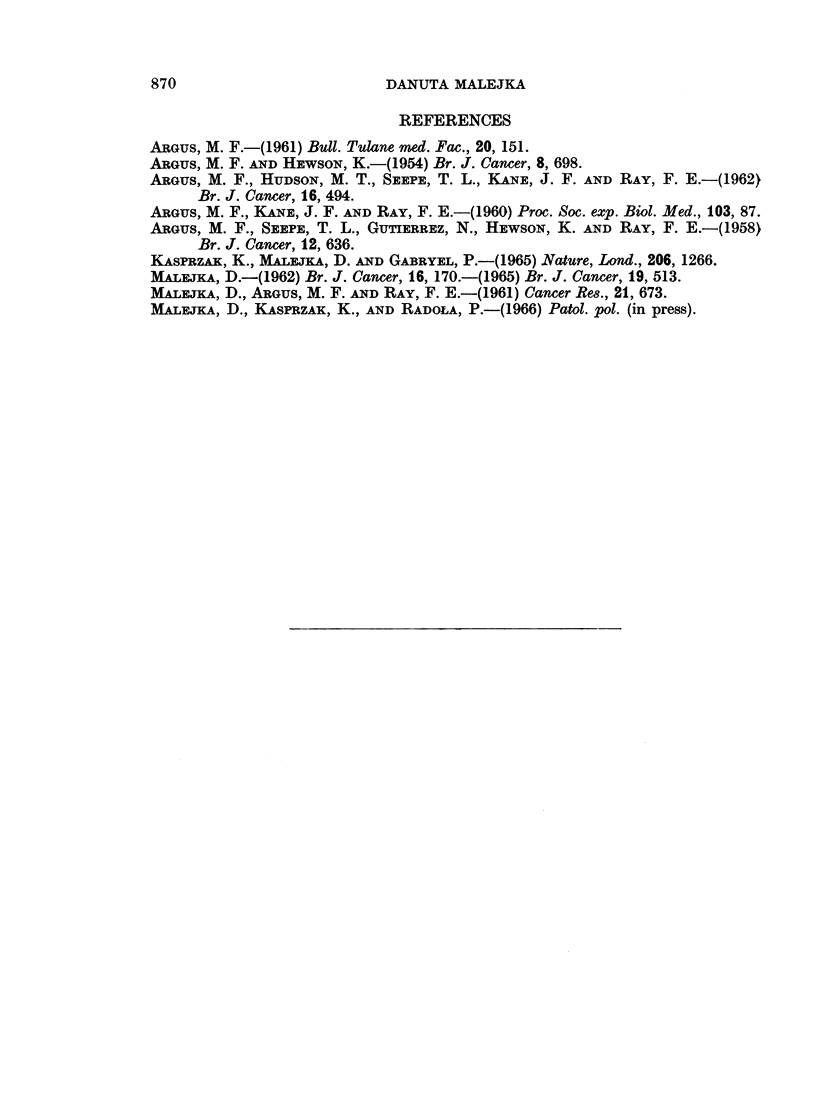

